# Classification and Severity Assessment of Obesity in Clinical Risk Prediction of Multimorbidity Trajectories

**DOI:** 10.1111/cob.70097

**Published:** 2026-07-15

**Authors:** Afua Ampadu‐Yeboah, Alistair Carr, Frederick Ho, Jason Gill, Naveed Sattar, Bhautesh Dinesh Jani

**Affiliations:** ^1^ School of Health and Wellbeing University of Glasgow Glasgow UK; ^2^ School of Cardiovascular & Metabolic Health University of Glasgow Glasgow UK

**Keywords:** body mass index, central adiposity, multimorbidity, obesity

## Abstract

Obesity is a key risk factor for chronic disease and multimorbidity, yet variation in how obesity is defined may influence risk estimation in clinical practise. Waist‐to‐hip ratio (WHR) and waist‐to‐height ratio (WHtR) are better at capturing fat distribution than body mass index (BMI) and may have a role in obesity related multimorbidity prediction. To examine multimorbidity risk across obesity groups classified using BMI in combination with WHR/WHtR. A UK Biobank cohort free of comorbidity at baseline (*n* = 179 876) was followed for incident first long‐term condition and multimorbidity (≥ 2 of 38 conditions) over a mean of 9.9 years. Six adiposity categories combining BMI with WHR/WHtR were analysed using Cox proportional hazards models. Subgroup analyses evaluated whether high central obesity (WHtR ≥ 0.6) provided additional risk stratification within BMI groups. Combined general with central obesity showed highest risk for the first condition (HR 1.11; 95% CI 1.087–1.138) and multimorbidity (HR 1.13; 95% CI 1.093–1.172). However, BMI‐stratified subgroup analyses, central obesity provided additional risk discrimination only among individuals in the overweight category (BMI 25–29.9 kg/m^2^), but not among those with BMI ≥ 30 kg/m^2^. Waist‐based measures improved risk identification in adults with overweight BMI, supporting the potential role of combined BMI‐waist approaches for earlier multimorbidity prevention.

## 1 Introduction

Multimorbidity, defined as the coexistence of two or more chronic conditions, is an escalating global health issue [1], particularly affecting over half of adults aged 65 years and older. Its prevalence is rising due to ageing populations and increasing obesity [2, 3], with global rates projected to reach 57% by 2050 [4]. In the UK, multimorbidity affects around a quarter of primary care patients and over half of older adults, disproportionately impacting socioeconomically disadvantaged groups and widening health inequalities [5, 6]. These trends highlight the need for prevention‐focused strategies, especially early interventions targeting modifiable risk factors such as obesity, to reduce future disease burden and healthcare costs.

Most multimorbidity research continues to rely solely on body mass index (BMI) as the measure of obesity. While BMI remains simple and widely applied in practise and policy, it does not distinguish fat from lean mass and fails to capture fat distribution [7]. This limitation may misclassify individuals, particularly those with normal BMI but excess central adiposity, a phenotype linked to elevated cardiometabolic risk [8]. Measures of central adiposity, including waist circumference (WC), waist‐to‐hip ratio (WHR), and waist‐to‐height ratio (WHtR), have gained recognition as stronger predictors of morbidity and mortality than BMI [9, 10]. WHR and WHtR, in particular, offer insight into visceral and ectopic liver adiposity, mediators driving disease [9]. A growing body of evidence indicates that these measures outperform BMI in predicting cardiovascular disease, diabetes, and premature mortality across diverse populations [10]. However, their predictive relevance for multimorbidity trajectories remains unclear.

Recent guidance from the 2025 *Lancet Diabetes & Endocrinology Commission* [11] advocates moving beyond BMI as a standalone indicator in all those with BMI < 40 (based on expert opinion), recommending its use primarily for population surveillance and encouraging the integration of central adiposity measures in both clinical and research contexts. However, multimorbidity research has been slow to adopt these recommendations, largely because it remains uncertain whether the inclusion of central adiposity measures over and above BMI adds meaningful predictive value for multimorbidity risk. The predictive ability of WHR, WHtR, or WC in combination with BMI has not been well evaluated. Furthermore, most existing multimorbidity studies [12, 13, 14] recruited participants who already have at least one long‐term condition (LTC) at baseline, making it difficult to isolate obesity's role in initiating multimorbidity rather than the increased risk that naturally follows from already having a first condition [15].

No prior study has systematically compared obesity classifications that jointly incorporate general obesity (BMI) and central adiposity within the same disease‐free cohort to evaluate their relative and combined associations with multimorbidity onset and burden, and done so by differing BMI subgroups. Addressing this gap is crucial for improving risk stratification and informing the development of targeted prevention and treatment strategies, especially in an era of newer, costly weight loss medications. Determining whether adding measures of WC, WHR or WHtR to BMI improves the assessment of multimorbidity risk has direct implications for clinical decision‐making and public health prioritisation.

Accordingly, this study aims to assess whether the risk of multimorbidity differs depending on how obesity is defined, using BMI and its combination with WHR/WHtR, in a large disease‐free cohort from the UK Biobank. This study further examines whether higher levels of central adiposity provide additional risk stratification for multimorbidity within BMI categories.

## 2 Materials and Methods

### 2.1 Study Design

This study employed a longitudinal cohort design using data from the UK Biobank to investigate associations between obesity and risk of multimorbidity development. Participants were followed from a disease‐free baseline to the incidence of (1) a first chronic condition and (2) subsequent multimorbidity.

### 2.2 Data Source

The UK Biobank is a large‐scale prospective health resource comprising over 500 000 participants aged 37–73 years recruited between 2006 and 2010 [16]. Baseline assessments included questionnaires, physical measurements, and biological samples, with subsequent linkage to hospital admissions, cancer registries, primary care, and mortality data [17]. Ethical approval was obtained from the North West Multi‐centre Research Ethics Committee (REC reference: 11/NW/0382; renewed 16/NW/0274), and all participants provided informed consent. Access for this study was granted under application ID 14151 [18].

### 2.3 Study Population

Participants were eligible if they were disease‐free at baseline (defined as the absence of any LTCs from the list of LTCs used in Barnett et al.'s [19] multimorbidity study, as described below) and had complete exposure and confounder data.

To derive the analytical cohort, we merged participants' baseline records with the First Occurrence and Cancer datasets using the unique participant ID (eid). These linked datasets were used to compile a unified disease occurrence file based on Barnett's list of long‐term conditions. Participants who had any recorded chronic condition prior to their baseline assessment were excluded. This process produced a disease‐free cohort at baseline, suitable for prospective analyses of incident multimorbidity.

### 2.4 Exposure Variables

#### 2.4.1 Obesity and Adiposity Categories

Obesity status was defined using body mass index (BMI), waist‐to‐hip ratio (WHR), and waist‐to‐height ratio (WHtR), measured at baseline by trained UK Biobank staff [17]. BMI was calculated as weight in kilograms divided by height in metres squared (kg/m^2^), and participants were classified as having normal weight (BMI 18.5–24.9 kg/m^2^), being in the overweight category (BMI 25.0–29.9 kg/m^2^), or having obesity (BMI ≥ 30.0 kg/m^2^) according to World Health Organisation (WHO) guidelines [20]. WHR was computed by dividing waist circumference by hip circumference, with obesity defined using WHO thresholds [21] of WHR ≥ 0.90 for men and ≥ 0.85 for women [22]. WHtR was calculated as waist circumference divided by height, and a WHtR ≥ 0.50, as recommended by the UK's National Institute for Health and Care Excellence (NICE), was used to define central obesity in both sexes [23].

For this analysis, BMI, WHR, and WHtR were combined to create six mutually exclusive adiposity categories, where each participant belonged to only one group. These were: (1) normal BMI with no central obesity (BMI 18.5–24.9, WHtR < 0.50, and WHR < 0.85 for women or < 0.90 for men); (2) normal BMI with central obesity (BMI 18.5–24.9 and central obesity defined by WHR and/or WHtR thresholds); (3) overweight BMI with no central obesity (BMI 25.0–29.9, WHtR < 0.50, and WHR below sex‐specific obesity thresholds); (4) overweight BMI with central obesity (BMI 25.0–29.9 and WHR ≥ 0.85 for women or ≥ 0.90 for men and/or WHtR ≥ 0.50); (5) obesity BMI with no central obesity (BMI ≥ 30.0 but not meeting central obesity thresholds); and (6) obesity BMI with central obesity (BMI ≥ 30.0 and central obesity defined by WHR and/or WHtR criteria).

These categories were created to reflect obesity driven by general adiposity, central adiposity or both, while preventing overlap across classifications. This enabled assessment of whether incorporating central adiposity measures alongside BMI improved the prediction of multimorbidity trajectories compared to the use of BMI alone.

### 2.5 Outcome Variables

Multimorbidity was defined as the incidence of two or more chronic conditions from a total of 40 conditions. These were selected based on the list developed by Barnett et al., which is widely used in multimorbidity research [19]. Two conditions (constipation and learning disability) were excluded due to data unavailability. Cancer was treated as a single condition, excluding benign, in situ, uncertain, and non‐melanoma skin cancers. Disease incidence was ascertained from the UK Biobank First Occurrence and Cancer datasets, coded using ICD‐10 and Read codes [24, 25].

### 2.6 Statistical Analysis

Baseline characteristics were summarised using means and standard deviations (SD) for continuous variables, and frequencies and percentages (%) for categorical variables, across adiposity categories. Cox proportional hazards regression models were used to estimate hazard ratios (HRs) and 95% confidence intervals (CIs) for the association of the six adiposity exposure categories with the outcomes (1) first condition and (2) multimorbidity (≥two conditions). Both unadjusted and adjusted models were fitted, with adjusted models controlling for demographic, lifestyle, and socioeconomic factors selected based on prior evidence (age, sex, ethnicity, smoking, alcohol intake, physical activity, household income, and Townsend deprivation quintiles). The Townsend deprivation index is an area‐based measure of socioeconomic deprivation derived from census data (including unemployment, non‐car ownership, non‐home ownership, and household overcrowding), and was categorised into quintiles, with higher quintiles indicating greater deprivation [26]. Alcohol consumption categories were derived from self‐reported drinking status and weekly alcohol units, and grouped into non‐, moderate‐, and higher‐risk consumption according to UK guideline thresholds. Physical activity was assessed using an IPAQ‐based questionnaire and categorised into low, moderate, and high activity based on MET‐minutes per week. Models were stratified by sex and 10‐year age bands to account for non‐proportional hazards, as the proportional hazards assumption was violated. Disease accumulation patterns and multimorbidity trajectories were summarised using descriptive statistics and heatmaps. All analyses were conducted using R version 4.5.1.

#### 2.6.1 Subgroup Analysis

Additional adiposity exposure categories analysed in subgroup analyses were to examine whether obesity defined with higher thresholds of central obesity (WHtR/WHR) conferred additional risk for developing multimorbidity relative to the standard thresholds analysed in the main analysis. In line with WHO and NICE recommendations [21, 23, 27], a WHR ≥ 1.0 and a WHtR ≥ 0.60 were used to denote high central obesity. Within the BMI obesity with central obesity category, participants were further classified into four mutually exclusive groups: (1) Class I obesity (BMI 30.0–34.9) with no high central obesity (WHtR 0.50–0.59 and WHR < 1.0); (2) Class I obesity with high central obesity (WHtR ≥ 0.60 and/or WHR ≥ 1.0); (3) Class II/III obesity (BMI ≥ 35.0) with no high central obesity; and (4) Class II/III obesity with high central obesity. For the BMI overweight with central obesity group, two additional subgroups were created: (1) overweight (BMI 25.0–29.9) with no high central obesity, and (2) overweight with high central obesity (WHtR ≥ 0.60 and/or WHR ≥ 1.0). Cox regression models stratified by sex and age band were again applied to estimate HRs and 95% CIs for the incidence of a first condition and multimorbidity within these refined categories.

## 3 Results

### 3.1 Descriptive Statistics

Out of the UK Biobank population (*n* = 502 538), a total of 179 876 participants who were disease‐free at baseline and had complete adiposity data were included in the analytical cohort. Table 1 presents the baseline characteristics of participants across the six adiposity groups. The most common adiposity categories were overweight BMI with central obesity (*n* = 63 695; 35.4%), followed by those with normal BMI with no central obesity (*n* = 50 882; 28.3%), and obesity BMI with central obesity (*n* = 33 570; 18.7%). Fewer participants belonged to the normal BMI with central obesity (*n* = 17 314; 9.6%) and overweight BMI without central obesity (*n* = 14 198; 7.9%), while only 217 participants (0.1%) had obesity BMI without central obesity.

**TABLE 1:** Baseline characteristics by adiposity group values are *n* (%) unless otherwise stated.

Characteristic/level	Normal BMI + no central obesity	Normal BMI + central obesity	Overweight BMI + no central obesity	Overweight BMI + central obesity	Obesity BMI + no central obesity	Obesity BMI + central obesity
*n* (%)	50 882 (28.3)	17 314 (9.63)	14 198 (7.9)	63 695 (35.4)	217 (0.12)	33 570 (18.7)
Age (mean (SD))	53.60 (8.08)	56.32 (8.00)	53.10 (8.10)	55.72 (8.07)	51.21 (7.79)	54.77 (7.98)
Sex (%)—female	36 834 (72.4)	6895 (39.8)	10 070 (70.9)	24 622 (38.7)	191 (88.0)	16 669 (49.7)
Sex (%)—male	14 048 (27.6)	10 419 (60.2)	4128 (29.1)	39 073 (61.3)	26 (12.0)	16 901 (50.3)
BMI (mean (SD))	22.55 (1.54)	23.67 (1.13)	26.28 (1.05)	27.42 (1.37)	30.99 (0.92)	33.44 (3.43)
Waist‐hip ratio (mean (SD))	0.79 (0.05)	0.90 (0.05)	0.79 (0.05)	0.90 (0.06)	0.74 (0.05)	0.92 (0.08)
WHtR (mean (SD))	0.45 (0.03)	0.51 (0.02)	0.48 (0.02)	0.54 (0.03)	0.48 (0.02)	0.61 (0.05)
Smoking (%)—current	4064 (8.0)	2066 (11.9)	1061 (7.5)	6039 (9.5)	17 (7.8)	2886 (8.6)
Smoking (%)—never	33 226 (65.2)	9991 (57.6)	9138 (64.3)	36 315 (56.9)	148 (68.2)	19 122 (56.9)
Smoking (%)—previous	13 540 (26.6)	5184 (29.9)	3977 (28.0)	21 116 (33.1)	51 (23.5)	11 392 (33.9)
Alcohol grp (%)—non	34 760 (68.2)	9958 (57.4)	9540 (67.1)	34 593 (54.2)	164 (75.6)	20 413 (60.7)
Alcohol grp (%)—moderate	14 245 (28.0)	6039 (34.8)	4094 (28.8)	24 062 (37.7)	45 (20.7)	10 513 (31.3)
Alcohol grp (%)—high	1733 (3.4)	1219 (7.0)	536 (3.8)	4767 (7.5)	7 (3.2)	2469 (7.3)
Physical activity (%)—high	8703 (17.1)	2042 (11.8)	2511 (17.7)	8089 (12.7)	27 (12.4)	2585 (7.7)
Physical activity (%)—medium	39 119 (76.8)	13 733 (79.2)	10 901 (76.7)	50 255 (78.8)	167 (77.0)	26 289 (78.2)
Physical activity (%)—low	1027 (2.0)	484 (2.8)	286 (2.0)	1861 (2.9)	9 (4.1)	1495 (4.4)
Physical activity (%)—none	1518 (3.0)	756 (4.4)	408 (2.9)	2676 (4.2)	13 (6.0)	2623 (7.8)
Townsend quintiles (%)—0–20	11 560 (22.7)	3683 (21.2)	3242 (22.8)	13 641 (21.4)	41 (18.9)	6072 (18.1)
Townsend quintiles (%)—20–40	10 817 (21.2)	3508 (20.2)	3109 (21.9)	13 383 (21.0)	47 (21.7)	6432 (19.1)
Townsend quintiles (%)—40–60	10 376 (20.4)	3424 (19.8)	2963 (20.8)	12 867 (20.2)	43 (19.8)	6645 (19.8)
Townsend quintiles (%)—60–80	9788 (19.2)	3410 (19.7)	2719 (19.1)	12 597 (19.8)	32 (14.7)	6960 (20.7)
Townsend quintiles (%)—80–100	8365 (16.4)	3294 (19.0)	2172 (15.3)	11 204 (17.6)	53 (24.4)	7443 (22.2)
Ethnic (%)—Asian or Asian British	611 (1.2)	654 (3.8)	128 (0.9)	1499 (2.4)	< 5	552 (1.6)
Ethnic (%)—Black or Black British	532 (1.0)	188 (1.1)	325 (2.3)	1237 (1.9)	11 (5.1)	1245 (3.7)
Ethnic (%)—Chinese	392 (0.8)	147 (0.8)	30 (0.2)	196 (0.3)	< 5	32 (0.1)
Ethnic (%)—mixed	339 (0.7)	73 (0.4)	96 (0.7)	354 (0.6)	< 5	222 (0.7)
Ethnic (%)—other ethnic group	420 (0.8)	200 (1.2)	103 (0.7)	714 (1.1)	< 5	453 (1.3)
Ethnic (%)—white	48 497 (95.2)	15 977 (92.2)	13 490 (94.9)	59 462 (93.2)	195 (89.9)	30 893 (91.9)

*Note:* Means are presented with standard deviations in parentheses.

Normal BMI with no central obesity: BMI 18.5–24.9 kg/m^2^ with WHR < 0.90 (men) or < 0.85 (women), and/or WHtR < 0.5.

Normal BMI with central obesity: BMI 18.5–24.9 kg/m^2^ with WHR ≥ 0.90 (men) or ≥ 0.85 (women) and/or WHtR ≥ 0.5.

Overweight BMI with no central obesity: BMI 25–29.9 kg/m^2^ with WHR < 0.90 (men) or < 0.85 (women), and/or WHtR < 0.5.

Overweight BMI with central obeesity: BMI 25–29.9 kg/m^2^ with WHR ≥ 0.90 (men) or ≥ 0.85 (women) and/or WHtR ≥ 0.5.

Obesity BMI with no central obesity: BMI ≥ 30 kg/m^2^ with WHR < 0.90 (men) or < 0.85 (women), and/or WHtR < 0.5.

Obesity BMI with central obesity: BMI ≥ 30 kg/m^2^ with WHR ≥ 0.90 (men) or ≥ 0.85 (women) and/or WHtR ≥ 0.5.

Women represented 53% of the overall cohort, but sex distributions varied considerably by adiposity group. Females predominated among those with normal BMI with no central obesity (72.4%) and overweight BMI without central obesity (70.9%), whereas males were more prevalent among groups with central obesity, particularly those who were overweight with central obesity (61.3%) and normal BMI with central obesity (60.2%). The mean age of the cohort was approximately 55 years (SD ≈ 8), with slight variations across adiposity categories. Participants with central obesity, irrespective of BMI category, tended to be older than those without central obesity.

As expected, mean BMI increased progressively from normal BMI (22.6 kg/m^2^) to overweight (26.3 kg/m^2^) and obesity (33.4 kg/m^2^). Central adiposity measures showed similar trends, with mean waist‐to‐hip ratio (WHR) and waist‐to‐height ratio (WHtR) highest among participants with both general and central obesity (0.92 ± 0.08 and 0.61 ± 0.05, respectively). Behaviour and socioeconomic characteristics also differed by adiposity status: individuals with central obesity were more likely to be previous or current smokers, less physically active, and had lower household income compared to those without central obesity.

### 3.2 Outcome Events

Over a mean follow‐up of 9.9 (±4.0) years, 70 412 participants (39.1%) developed a first condition, and 31 515 (17.5%) progressed to multimorbidity (Table 2). The highest proportions of both outcomes were observed among participants classified with obesity by BMI with central obesity (with 46.8% developing a first condition and 24.1% progressing to multimorbidity), and in participants classified as being overweight by BMI with central obesity (with 42.0% experiencing a first condition and 19.6% developing multimorbidity). In contrast, those with normal BMI and no central obesity had the lowest incidence (31.8% first condition; 11.6% multimorbidity). Participants with normal BMI and central obesity demonstrated noticeably higher proportions of outcomes (40.0% and 18.2%, respectively) compared with those with normal BMI but no central obesity. The smallest group, obesity without central obesity (*n* = 217), displayed intermediate event proportions (34.1% first condition; 12.4% multimorbidity), though small numbers limit interpretation.

**TABLE 2:** Summary of outcome events by adiposity groups.

Adiposity group	Total (*n*)	First condition (*n*)	Multimorbidity (*n*)	First condition (%)	Multimorbidity (%)
Normal BMI + no central obesity	50 882	16 180	5902	31.8	11.6
Normal BMI + central obesity	17 314	6926	3151	40.0	18.2
Overweight BMI + no central obesity	14 198	4799	1803	33.8	12.7
Overweight + central obesity	63 695	26 752	12 484	42.0	19.6
Obesity + no central obesity	217	74	27	34.1	12.4
Obesity + central obesity	33 570	15 711	8090	46.8	24.1

*Note:* Normal BMI + no central obesity: BMI 18.5–24.9 kg/m^2^ with WHR < 0.90 (men) or < 0.85 (women), and/or WHtR < 0.5.

Normal BMI + central obesity: BMI 18.5–24.9 kg/m^2^ with WHR ≥ 0.90 (men) or ≥ 0.85 (women) and/or WHtR ≥ 0.5.

Overweight BMI + no central obesity: BMI 25–29.9 kg/m^2^ with WHR < 0.90 (men) or < 0.85 (women), and/or WHtR < 0.5.

Overweight BMI + central obeesity: BMI 25–29.9 kg/m^2^ with WHR ≥ 0.90 (men) or ≥ 0.85 (women) and/or WHtR ≥ 0.5.

Obesity BMI + no central obesity: BMI ≥ 30 kg/m^2^ with WHR < 0.90 (men) or < 0.85 (women), and/or WHtR < 0.5.

Obesity BMI + central obesity: BMI ≥ 30 kg/m^2^ with WHR ≥ 0.90 (men) or ≥ 0.85 (women) and/or WHtR ≥ 0.5.

Adjusted for: sex, ethnicity, smoking, alcohol intake, physical activity, household income, and Townsend deprivation quintiles.

### 3.3 Most Common Conditions

**FIGURE 1:**
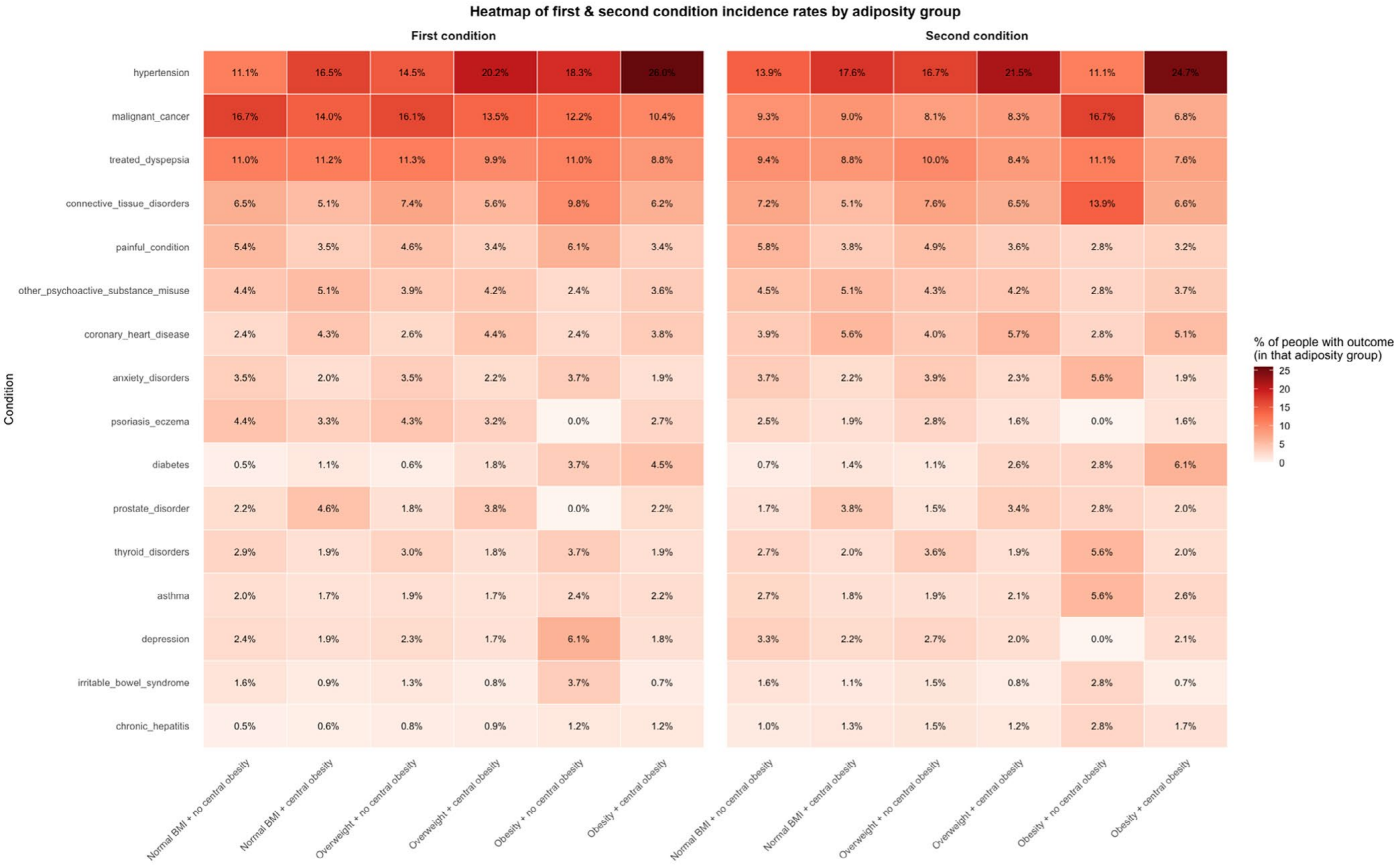
Heatmap of first & second condition incidence rates by adiposity group. Interpreting these percentages requires attention to the fact that we are examining the sequencing of conditions, not total disease frequency. The percentages in the heatmap represent the proportion of individuals within each adiposity group whose first & second recorded condition was a specific disease, among those who developed any condition at all. These do not reflect the overall incidence or risk of each disease in the adiposity groups. This heatmap illustrates, within each adiposity group, the most common first and second conditions rather than comparing absolute risk across groups. Normal BMI with no central obesity: BMI 18.5–24.9 kg/m^2^ with WHR < 0.90 (men) or < 0.85 (women), and/or WHtR < 0.5. Normal BMI with central obesity: BMI 18.5–24.9 kg/m^2^ with WHR ≥ 0.90 (men) or ≥ 0.85 (women) and/or WHtR ≥ 0.5. Overweight BMI with no central obesity: BMI 25–29.9 kg/m^2^ with WHR < 0.90 (men) or < 0.85 (women), and/or WHtR < 0.5. Overweight BMI with central obesity: BMI 25–29.9 kg/m^2^ with WHR ≥ 0.90 (men) or ≥ 0.85 (women) and/or WHtR ≥ 0.5. Obesity BMI with no central obesity: BMI ≥ 30 kg/m^2^ with WHR < 0.90 (men) or < 0.85 (women), and/or WHtR < 0.5. Obesity BMI with central obesity: BMI ≥ 30 kg/m^2^ with WHR ≥ 0.90 (men) or ≥ 0.85 (women) and/or WHtR ≥ 0.5.

Figure 1 presents the most common first and second chronic conditions (with the second condition contributing to multimorbidity status) across adiposity classifications. These patterns highlight variation in first and second chronic diseases between exposure groups.

Across adiposity categories, hypertension consistently emerged as the most common first condition, increasing from 11.1% in the normal BMI with no central obesity group to 26.0% in the general obesity with central obesity group. Malignant cancer and treated dyspepsia were also frequent first conditions across all categories, although their relative positions differed, with malignant cancer being more common in the lower‐adiposity groups (e.g., 16.7% in normal BMI with no central obesity), while hypertension predominated in groups with higher adiposity.

Several conditions showed more distinct distributions. Diabetes (including both type 1 and type 2) appeared as a common first condition almost exclusively within the obesity categories (3.7% in general obesity with no central obesity; 4.5% in general obesity with central obesity), remaining below 1% in all non‐obesity groups. Prostate disorder occurred more frequently in central‐obesity categories, and coronary heart disease appeared predominantly among individuals with central obesity across BMI strata.

Patterns for the most common second conditions largely mirrored those of the first. Hypertension again featured most prominently, ranging from 13.9% in normal BMI with no central obesity to 24.7% in general obesity with central obesity. Malignant cancer and treated dyspepsia continued to recur across all categories. Diabetes (including both type 1 and type 2) as a second condition was again most frequent within the obesity groups, while prostate disorder appeared mainly in the two central‐obesity categories. Coronary heart disease remained more apparent among individuals with central obesity, whereas other conditions, including anxiety disorders and asthma, displayed modest and relatively stable percentages across the adiposity spectrum.

### 3.4 Associations With First Condition and Multimorbidity

Table 3 presents the adjusted Cox regression results for the association between adiposity categories and the risk of developing a first chronic condition and subsequent multimorbidity.

**TABLE 3:** Adjusted hazard ratios for incident first condition and multimorbidity by adiposity group (*n* = 179 876).

Adiposity group	First condition		Multimorbidity	
	HR	95% CI	HR	95% CI
Normal BMI + no central obesity (*n* = 50 882)	Reference	—	Reference	—
Normal BMI + central obesity (*n* = 17 314)	1.01	0.984–1.043	1.00	0.958–1.049
Overweight + no central obesity (*n* = 14 198)	1.02	0.988–1.055	1.06	1.003–1.116
Overweight + central obesity (*n* = 63 695)	1.04	1.023–1.066	1.06	1.022–1.092
Obesity BMI + no central obesity (*n* = 217)	1.15	0.913–1.451	1.10	0.759–1.618
Obesity BMI + central obesity (*n* = 33 570)	1.11	1.087–1.138	1.13	1.093–1.172

*Note:* Normal BMI + no central obesity: BMI 18.5–24.9 kg/m^2^ with WHR < 0.90 (men) or < 0.85 (women), and/or WHtR < 0.5.

Normal BMI + central obesity: BMI 18.5–24.9 kg/m^2^ with WHR ≥ 0.90 (men) or ≥ 0.85 (women) and/or WHtR ≥ 0.5.

Overweight BMI + no central obesity: BMI 25–29.9 kg/m^2^ with WHR < 0.90 (men) or < 0.85 (women), and/or WHtR < 0.5.

Overweight BMI + central obeesity: BMI 25–29.9 kg/m^2^ with WHR ≥ 0.90 (men) or ≥ 0.85 (women) and/or WHtR ≥ 0.5.

Obesity BMI + no central obesity: BMI ≥ 30 kg/m^2^ with WHR < 0.90 (men) or < 0.85 (women), and/or WHtR < 0.5.

Obesity BMI + central obesity: BMI ≥ 30 kg/m^2^ with WHR ≥ 0.90 (men) or ≥ 0.85 (women) and/or WHtR ≥ 0.5.

Adjusted for: sex, ethnicity, smoking, alcohol intake, physical activity, household income, and Townsend deprivation quintiles.

Compared with the reference group (normal BMI + no central obesity), participants with normal BMI and central obesity showed no significant difference in risk for either first condition or multimorbidity. Those with overweight but no central obesity had a modestly higher risk of multimorbidity (HR = 1.06; 95% CI 1.003–1.116) but not for the first condition (HR = 1.02; 95% CI 0.988–1.055). Participants with overweight and central obesity showed significantly elevated risks for both outcomes, the first condition (HR = 1.04; 95% CI 1.023–1.066) and multimorbidity (HR = 1.06; 95% CI 1.022–1.092). Among those with obesity BMI and no central obesity, risk estimates were higher but imprecise due to the small sample size. Finally, the obesity BMI with central obesity group demonstrated the highest and statistically significant risks for both outcomes, the first condition (HR = 1.11; 95% CI 1.087–1.138) and multimorbidity (HR = 1.13; 95% CI 1.093–1.172).

### 3.5 Subgroup Analysis—Overweight and Obesity Categories

Adjusted hazard ratios (HRs) were used to compare the risk of developing a first condition and subsequent multimorbidity across combinations of BMI class and central obesity status within the overweight and obesity subgroups. Table 4 presents the adjusted Cox regression results for the overweight subgroup, and Table 5 presents results for the obesity subgroup, each reporting associations with the first condition and multimorbidity.

**TABLE 4:** Subgroup analysis among participants who were overweight (BMI) with central obesity (*n* = 63 695): Adjusted hazard ratios for incident first condition and multimorbidity by central obesity.

Adiposity group	First condition		Multimorbidity	
	HR	95% CI	HR	95% CI
No high central obesity (*n* = 58 196)	Reference	—	Reference	—
High central obesity (*n* = 5499)	1.06	1.021–1.108	1.15	1.086–1.214

*Note:* No high central obesity: WHtR 0.50–0.59; WHR 0.85–0.99.

High central obesity: WHtR ≥ 0.60; WHR ≥ 1.

**TABLE 5:** Subgroup analysis among participants with general obesity (BMI) and central obesity (*n* = 33 570): Adjusted hazard ratios for incident first condition and multimorbidity by central obesity.

Adiposity group	First condition		Multimorbidity	
	HR	95% CI	HR	95% CI
Class I + No high central obesity (*n* = 13 520)	Reference	—	Reference	—
Class I + High central obesity (*n* = 12 198)	0.99	0.95–1.03	1.00	0.95–1.06
Class II/III + No high central obesity (*n* = 627)	1.00	0.89–1.14	0.96	0.79–1.16
Class II/III + High central obesity (*n* = 7225)	1.05	1.00–1.09	1.05	0.99–1.11

*Note:* Class I: BMI 30–34.9 kg/m^2^.

Class II/III: BMI ≥ 35 kg/m^2^.

Among participants who were overweight, those with high central obesity had significantly higher risks for both outcomes compared with their counterparts without high central obesity. The adjusted hazard ratio (HR) for the first condition was 1.06 (95% CI: 1.021–1.108), while for multimorbidity, it was 1.15 (95% CI: 1.086–1.214).

Within the obesity subgroup, no clear differences were observed across classes after adjustment. For the first condition, HRs ranged from 0.99 (95% CI: 0.95–1.03) in Class I with high central obesity to 1.05 (95% CI: 1.00–1.09) in Class II/III with high central obesity. A similar pattern was seen for multimorbidity, with HRs ranging from 0.96 (95% CI: 0.79–1.16) to 1.05 (95% CI: 0.99–1.11), and none reaching statistical significance.

## 4 Discussion

This study examined whether the definition and classification of obesity, based on BMI in combination with central adiposity measures, affects its observed association with incident chronic conditions and progression to multimorbidity among disease‐free adults at baseline.

### 4.1 Principal Findings

Four key findings emerged. First, the overweight with central obesity group was the largest group and therefore contributed the greatest number of incident cases, but risk was highest among individuals with general obesity and central obesity, with 46.8% developing a first condition and 24.1% progressing to multimorbidity. Second, hypertension, malignant cancer, and treated dyspepsia were the most common first and second conditions, with distinct patterns across adiposity groups: diabetes was more common in BMI‐defined obesity, coronary heart disease was more common in groups with central obesity, and prostate disorders were confined to the central‐obesity categories of normal BMI and overweight. Third, adjusted Cox models showed the highest risks in the general obesity with central obesity category (HRs 1.11 for first condition and 1.13 for multimorbidity), followed by overweight with central obesity (HRs 1.04 and 1.06), with other groups showing estimates close to the null. Finally, subgroup analyses indicated that high central obesity added meaningful risk only within the overweight subgroup, while effects within the obesity subgroup were small and imprecise.

Taken together, these findings indicate that (i) central adiposity contributes to, but does not substantially alter, overall risk prediction for multimorbidity; (ii) incorporating central measures appears most informative within the overweight range, where BMI alone may understate risk; and (iii) examining adiposity patterns provides insight into condition‐specific trajectories and potential phenotypic clustering of multimorbidity.

### 4.2 Interpretation in the Context of Prior Evidence

The present findings contribute new evidence to whether central obesity measures in combination with BMI improve multimorbidity risk prediction. Consistent with the recent analysis by Ho et al. [22], which examined 78 incident conditions and multiple long‐term conditions in UK cohorts and concluded that waist‐based measures (WHR, WHtR) provided little additional predictive value once BMI reached ≥ 30 kg/m^2^. Similarly, in our study, adjusted hazards for both the first condition and multimorbidity among obesity subgroups were small, non‐significant, and imprecise. Only Class II/III obesity with high central obesity showed a modest elevation in risk. These findings reinforce the view that, at higher BMI levels, BMI captures most of the cardiometabolic risk, reducing the incremental utility of central obesity measures.

Our results demonstrate that central obesity meaningfully differentiates risk within the overweight range, consistent with Ho et al. [22] and earlier work identifying individuals with normal weight but elevated obesity‐related metabolic risk [28, 29]. Participants in the overweight category with high central obesity exhibited significantly higher hazards for both outcomes than those without high central obesity, indicating that abdominal fat distribution is most informative where BMI may underestimate metabolic risk [9]. This aligns with mechanistic literature emphasising visceral adiposity as a key driver of insulin resistance, systemic inflammation, and vascular dysfunction [30, 31].

Disease patterns observed within adiposity categories further explains some of the potential mechanisms involved. Diabetes appeared solely within obesity groups, supporting strong evidence linking impaired glucose regulation predominantly to higher total adiposity rather than to fat distribution alone [32]. Conversely, hypertension was present across all groups but highest in central‐obesity categories, aligning with evidence that visceral fat promotes sympathetic activation, RAAS upregulation, and endothelial dysfunction [33]. The high prevalence of coronary heart disease in central‐obesity groups, both as a first and second condition, supports the well‐established link between abdominal fat deposition, dyslipidaemia, and atherogenesis [34]. Finally, the exclusive appearance of prostate disorders in central‐obesity but without BMI‐defined obesity echoes findings by Hurwitz et al. and Lavalette et al., suggesting waist‐based measures capture urogenital risk pathways missed by BMI [35, 36].

### 4.3 Strengths and Limitations

Strengths include: (i) a large cohort free of LTC at baseline, enabling clear temporal ordering; (ii) the use of mutually exclusive adiposity groups that combine general adiposity (BMI‐defined) and central adiposity; (iii) nurse‐measured anthropometry; (iv) consistent patterns across descriptive, heat‐map, and model‐based analyses; and (v) analysis of both first condition and progression to multimorbidity, and near complete follow‐up using linked administrative records.

There are some notable limitations of this study. First, adiposity was measured once at baseline; unmeasured weight change may have led to exposure misclassification and dilution of effects. Second, selection into the UK Biobank and the restriction to disease‐free participants at baseline likely resulted in lower absolute risks and healthier profiles overall. This reflects the well‐documented healthy volunteer effec*t*, whereby UK Biobank participants tend to be healthier, more health‐conscious, and more socioeconomically advantaged than the general population [37, 38]. Consequently, estimates may underestimate true population risks and limit generalisability, particularly for underrepresented ethnic and socioeconomically disadvantaged groups. Third, the obesity + no central obesity group was very small, yielding wide confidence intervals, which made it difficult to estimate multimorbidity risk for individuals in that category.

Fourth, the analysis applied standard BMI and central adiposity thresholds that are primarily derived from White European populations. These thresholds may not be appropriate for all ethnic groups, particularly South Asian populations, who have been shown to experience higher cardiometabolic risk at lower levels of adiposity compared with White populations [39, 40]. Given that the UK Biobank cohort is predominantly of White ethnicity [38], the generalisability of these findings to more ethnically diverse populations is limited, and the magnitude of risk associated with central adiposity may differ across ethnic groups.

### 4.4 Clinical and Public Health Implications

These findings suggest that incorporating a central obesity measure alongside BMI may improve early risk identification, particularly among individuals with overweight, where BMI alone underestimates susceptibility to multimorbidity. Because this group is large and contributes meaningfully to future incidence, targeted multimorbidity prevention strategies using both BMI and a simple waist‐based metric could support earlier intervention. While central obesity measures added little discriminatory value at higher BMI levels, they offered clearer differentiation of cardiometabolic and condition‐specific patterns, highlighting their utility in risk stratification and prevention planning.

### 4.5 Directions for Future Research

Future work should: (i) incorporate repeated adiposity measurements to capture weight trajectories and central fat dynamics; (ii) validate these findings in more diverse, representative cohorts, including populations for whom different adiposity thresholds may apply; and (iii) take a life‐course perspective to identify upstream determinants of multimorbidity accumulation among those with central adiposity. Trials and implementation studies testing dual‐measure screening and eligibility rules for interventions would provide the strongest evidence for changing practise.

## 5 Conclusions

This study demonstrates that how obesity is defined has important implications for assessing future multimorbidity risk and the identification of vulnerable groups. Although adding central obesity measures to BMI did not substantially increase risk estimates among individuals already living with obesity, the subgroup analysis showed clear added value within the overweight category. Given that an estimated 2.5 billion people, around 31% of the world's population, were classified as overweight in 2022 [27], improving multimorbidity risk identification in this group is of substantial global health relevance. If waist‐based measures in combination with BMI more accurately flag higher‐risk overweight individuals, our findings support recent recommendations, such as those from the Lancet Commission [11], to adopt a dual‐metric approach to obesity assessment.

Incorporating a simple central obesity measure alongside BMI has the potential to improve early identification of at‐risk individuals and more accurately capture the scale and distribution of multimorbidity risk. As new obesity therapies and prevention strategies become increasingly available, optimising obesity classification in a clinical practise is a timely public health priority to support equitable, targeted, and effective intervention.

## Author Contributions

Afua Ampadu‐Yeboah conducted the data analysis and literature review, interpreted the findings, and drafted the manuscript. Bhautesh Dinesh Jani conceived the study and research topic, and together with Alistair Carr contributed to study design, interpretation of the results, and critical revision of the manuscript for important intellectual content. Frederick Ho, Naveed Sattar, and Jason Gill contributed to study design, provided methodological input, and reviewed the manuscript. All authors contributed to manuscript development, approved the final version for submission, and agreed to be accountable for all aspects of the work.

## Conflicts of Interest

Prof. Naveed Sattar has consulted for and/or received speaker honoraria from AbbVie, Amgen, AstraZeneca, Boehringer Ingelheim, Carmot Therapeutics, Eli Lilly, Gan & Lee, GlaxoSmithKline, Hanmi Pharmaceuticals, Kailera, Mass Medicines, Menarini‐Ricerche, Metsera, Novo Nordisk, Pfizer, Regeneron, Roche, UCB Pharma, and Verdiva Bio; and received grant support paid to his University from AstraZeneca, Boehringer Ingelheim, Novartis, and Roche outside the submitted work.

## Data Availability

The data that support the findings of this study are available from UK Biobank. Restrictions apply to the availability of these data, which were used under license for this study. Data are available from the author(s) with the permission of UK Biobank.
